# Supplementary material for: Thylakoid‐integrated recombinant Hcf106 participates in the chloroplast twin arginine transport system
Source: Plant Direct. 2018 Oct 24;2(10):e00090. doi: 10.1002/pld3.90 (PMC6508782; doi:10.1002/pld3.90)
Supplement: Supplementary file 1 — Figure S1. Hcf106 with cysteine substitutions in the (A) N‐terminus, (B) TMD, (C) hinge integrates into thylakoid and is resistant to alkaline extraction. Figure S2. Quantification of Hcf106 dimer formation in the TMD and APH. Figure S3. Most Hcf106 dimers disappear in the presence of the reducing agent, dithiothreitol (DTT). [file PLD3-2-e00090-s001.pdf]

Ma, Q., Fite, K., New, C.P., and Dabney-Smith, C., Thylakoid-integrated recombinant Hcf106 participates in the chloroplast Twin Arginine Transport (cpTat) system as revealed by crosslinking.

Supplementary materials

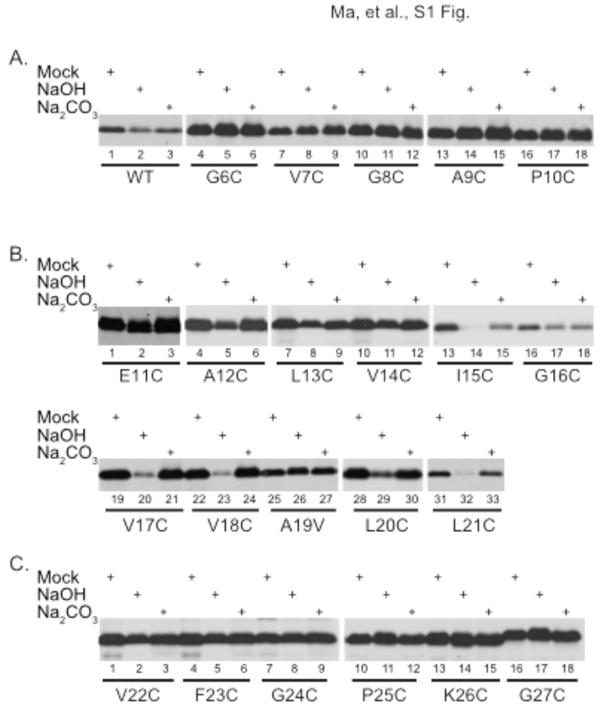

S1 Fig. Hcf106 with cysteine substitutions in the (A) N-terminus, (B) TMD, (C) hinge integrates into thylakoid and is resistant to alkaline extraction. In vitro translated Cys-substituted Hcf106 variants from N-terminus to hinge region were incubated with isolated thylakoids in protein integration assay. The thylakoids then subjected to alkaline extraction using either 0.2 M carbonate buffer (pH 9.0) or 0.1 M NaOH (pH 11.0). The wild-type Hcf106 was used as a control. Single Cys substitutions in Hcf106 are indicated in the top of the panels. Samples were analyzed by SDS-PAGE and fluorography. Gels in all panels are representative of at least three separate experiments. Thick white space between panels indicates separate gels.

Ma, Q., Fite, K., New, C.P., and Dabney-Smith, C., Thylakoid-integrated recombinant Hcf106 participates in the chloroplast Twin Arginine Transport (cpTat) system as revealed by crosslinking.

Ma, et al., S2 Fig

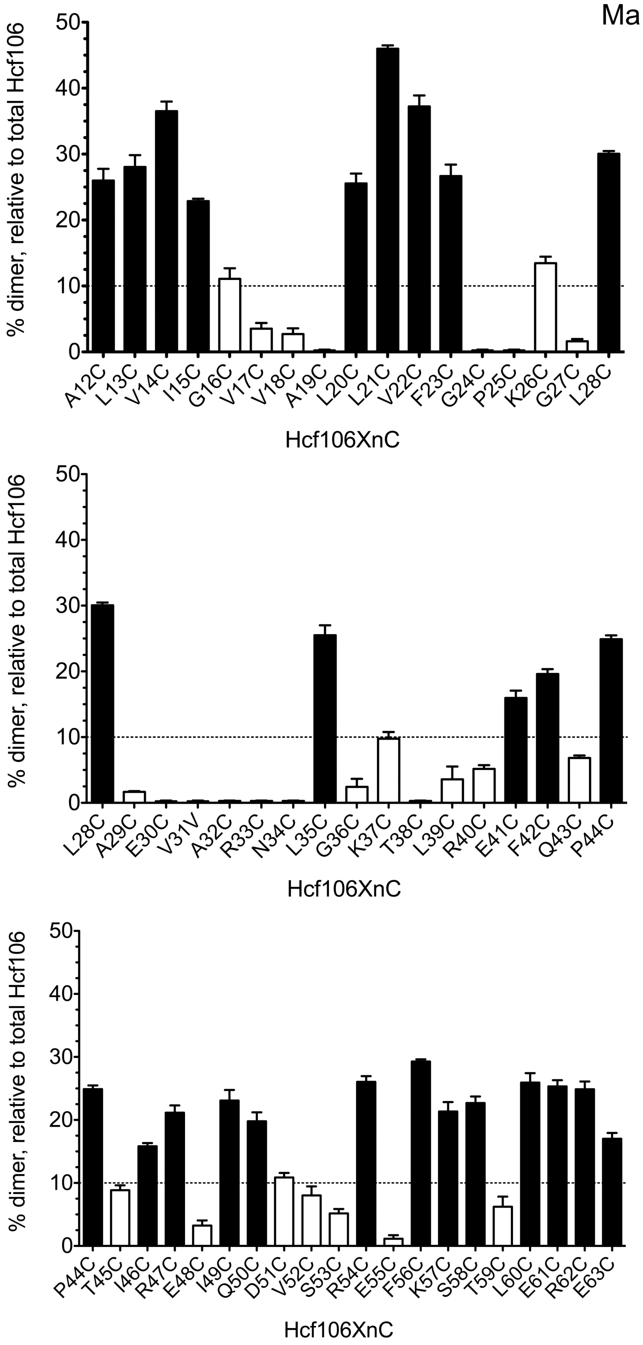

S2 Fig. Quantification of Hcf106 dimer formation in the TMD and APH. The proportion of cross-linking for each Hcf106 Cys-substitution was calculated based on the intensity of the dimer band divided by the intensity of the dimer plus monomer bands. Intensity was measured using ImageJ as described in the Materials and Methods. Data presented are the means ± S.E. of at least three separate experiments. Dark bars indicate the dimer is greater than 10% of total added Hcf106, while white bars indicate ≥10% of total added Hcf106.

Ma, et al., S3 Fig.

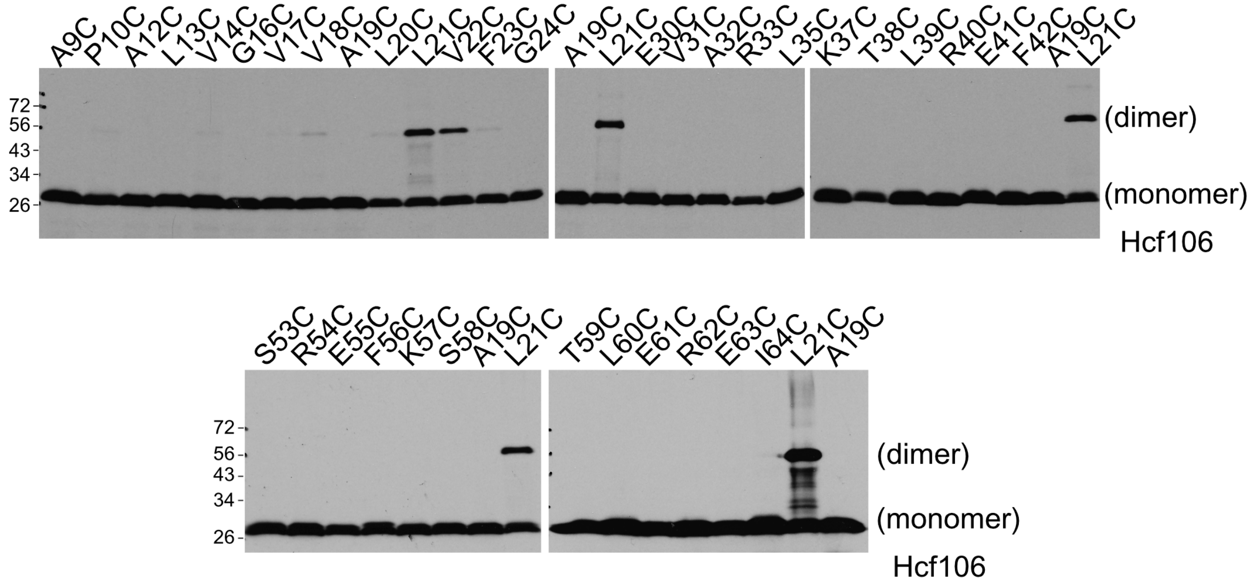

S3 Fig. Most Hcf106 dimers disappear in the presence of the reducing agent, dithiothreitol (DTT). Hcf106 single Cys- cross-linking samples were treated with DTT in a reducing sample buffer as described in Materials and Methods. Most of the Hcf106 dimers were reduced by addition of DTT except L21C and V22C, which still show the 56 kDa dimer bands. Gels in panels are representative of at least three separate experiments. Thick white space between panels indicates separate gels.
