# Supplementary material for: Thylakoid‐integrated recombinant Hcf106 participates in the chloroplast twin arginine transport system
Source: Plant Direct. 2018 Oct 24;2(10):e00090. doi: 10.1002/pld3.90 (PMC6508782; doi:10.1002/pld3.90)
Supplement: Supplementary file 2 [file PLD3-2-e00090-s002.pdf]

MIAMI UNIVERSITY
OXFORD, OH • EST. 1809

CAROLE DABNEY-SMITH
Associate PROFESSOR
CHEMISTRY AND BIOCHEMISTRY
132 HUGHES LABORATORIES
OXFORD, OHIO 45056
(513) 529-8091
adabney@MiamiOH.edu

October 2, 2018

Dear Editor,

We are submitting a revised original research article titled '**Thylakoid-integrated recombinant Hcf106 participates in the chloroplast Twin Arginine Transport (cpTat) system'** by Qianqian Ma, Kristen Fite, Christopher Paul New, and Carole Dabney-Smith for consideration for consideration by *Plant Direct*. This manuscript was originally submitted with the same title and has manuscript number **2018-00189**. We thank you for your time and effort in handling our manuscript. We would also thank the reviewers for such careful attention to our manuscript. Their comments, suggestions, and feedback are much appreciated and helped make the manuscript stronger. We have addressed the Reviewer's comments and suggestions and have included a "tracked changes" version to make it easier for the reviewers to see changes that were made. Our responses below represent those changes in the manuscript.

Reviewer #1:
This manuscript investigated the role of Hcf106 in cpTat receptor complex oligomerization and stability. The authors made systematic cysteine substitutions of Hcf106 in positions from the N-terminal transmembrane domain to the end of the predicted amphipathic helix of the protein, and identified the locations of amino acids in Hcf106 that were critical for interacting with cpTatC. They also showed that integrated Hcf106 could interact with the precursor signal peptide domain and imported cpTatC.

1. Line 56, "Tat translocase" was mentioned directly without defining it;
We apologize for the oversight. A brief description of the protein subunits of the Tat translocase complex was added to the sentence.

New Lines 56-60

2. Line 58, "Receptor complex" was mentioned directly without defining it; is it the Tat complex?
We apologize for the lack of clarity. Yes, we did mean the Tat complex. We have made the correction.

3. Line 84, "of" is missing between "exploration" and "Hcf106";
Corrected.

4. Line 84, maybe add more information about "cysteine substitution" and why cysteine is chosen.
We have added a description of cysteine substitution to highlight prior use of the technique in plant and bacterial Tat studies.

New Lines 87-104

5. Fig1. What is the meaning of dark circle around P44?

CAROLE DABNEY-SMITH
Associate PROFESSOR
CHEMISTRY AND BIOCHEMISTRY
132 HUGHES LABORATORIES
OXFORD, OHIO 45056
(513) 529-8091
adabney@MiamiOH.edu

We apologize for the oversight. The P44 of Hcf106 was filled in black because it was chosen as the arbitrary break point for the helical wheel projections in Figure 5. We have added the information to the legend for Figure 1.

New lines 801-802

6. Fig2, where are the loading controls of equal amount of Hcf106 variants and equal amounts of thylakoids? Where is the negative control of this experiment, e.g. some proteins that are not expected to integrate with the Tat complex?
Yes, we normalized the samples to total protein content and loaded equal amounts on the gels. We have clarified that information in the Materials and Methods and in the legend to Figure 2.

New lines 193-195 and 806-807:

7. Fig 3. "The amount of Hcf1061-107 added to thylakoid corresponds to dilutions of the in vitro translation reaction (i.e., 1:60, 1:12, 1:6, and 1:3)." It would be better to use a loading control to quantify the amount of Hcf1061-107. With the increased amount of truncated Hcf106, why the bands using anti-Hcf106 didn't increase?
Samples from thylakoids after $Hcf106_{1-107}$ integration that were loaded on the gel were normalized to equal protein loading as in Figure 2. The band of anti-Hcf106 doesn't increase because we are actually detecting all Hcf106 in thylakoid, not only the in vitro translated $Hcf106_{1-107}$.

It would be better to have a positive control using full length Hcf106 and a negative control from Fig 2, one of the variants that cannot integrate to the complex.
In this experiment, using full-length proteins as controls yields a result difficult to interpret because we lack the visual that a difference in size due to the presence of the truncation provides. Using equal protein loading allows us to visually distinguish the contributions to the different complexes to the presence of the truncated $Hcf106_{1-107}$.

8. Fig 4, where is the WT control?
Hcf106 lacks any cysteines, and we used the lack of dimer formation with the A19C variant to indicate the negative control of no dimer formation.

9. Fig 5, "The shading represents the hydrophobicity of the amino acid, hydrophilic amino acids are more lightly shaded". This is confusing, did you mean dark circles and grey circles?
We apologize for the confusion. We have modified the text to be clearer.

New lines: 838-839

10. Fig. 6, to see the effects of double-cysteine substitutions, it is better to load double and single cysteine substitutions on the same gel for comparison?
We do not disagree with the reviewer; however, the reviewer is referred to Figure 4 to see the dimer formation of the single cysteine variants. We have replaced the images in Figure 4 with images encompassing more of the field of the gel to aid in the comparison.

CAROLE DABNEY-SMITH
Associate PROFESSOR
CHEMISTRY AND BIOCHEMISTRY
132 HUGHES LABORATORIES
OXFORD, OHIO 45056
(513) 529-8091
adabney@MiamiOH.edu

11. Fig s1, "Single Cys substitutions in Hcf106 are indicated in the top of the panels." Did you mean at the "bottom" of the panels?
Yes, we apologize for the error and have corrected the legend to reflect the correct location of the labels.

12. Fig s3, the results of L20C weren't consistent in the first and third gels on the top, why?
We thank the reviewer for bringing this to our attention. We used A19C and L21C as negative and positive controls for dimerization. The L20C on the third panel is a typo and has been corrected to be L21C in the figure.

13. Line 415-417 are confusing and need revision.
We apologize for the lack of clarity. We have revised the lines.

New lines: 453-457

14. Line 501-502, "We also identified residues in Hcf106 that are important to receptor complex assembly", this statement isn't sound. The data just showed that these variants have reduced integration into the complex but didn't show it affected complex assembly.
We agree with the reviewer. We have clarified the statement to reflect that the ability of the Hcf106 to integrate into the complex was hindered. Please see lines 562-563.

15. In the discussion section, when the authors discussed the results from this manuscript, it would be better to mention Fig numbers of the results.
Thank you for the suggestion. We have incorporated references to figures into the discussion.

16. Line 532-533, could you do complementation in TatB mutant background?
Genetic complementations are possible, but *hcf106* mutants in *Arabidopsis thaliana* are seedling lethal making biochemical complementation unfeasible. Please see lines 533-535.

17. How do Hcf106 with cysteine substitutions affect the function and efficiency of the Tat complex?
Currently, due to the presence of endogenous Hcf106 in the receptor complex, we can deduce that the presence of the cysteine variants in the receptor complex does not severely inhibit complex functionality. Please see lines 594-601.

Reviewer #2:

In this manuscript entitled "Thylakoid-integrated recombinant Hcf106 participates in the chloroplast Twin Arginine Transport (cpTat) system" by Ma et al. authors described the integration of truncated Hcf106 into the thylakoid membranes and showed that the truncated Hcf106 forms a part of receptor complex, interacts with Tha4 (weekly) and cpTatC. This truncated form of Hcf106 interacts with the cysteine scanning mutagenesis Hcf106 was very informative, thought a similar work has been done in a bacterial system.

CAROLE DABNEY-SMITH
Associate PROFESSOR
CHEMISTRY AND BIOCHEMISTRY
132 HUGHES LABORATORIES
OXFORD, OHIO 45056
(513) 529-8091
adabney@MiamiOH.edu

The manuscript is well written and all the results are systematically summarized in the discussion section. The gels are clean and appropriate controls were included in the study. Many experiments were repeated multiple times.

Comments
1. The title is confusing. From the title is appears the truncated Hcf106 can support Tat transport in the thylakoid system.

We apologize for the confusion. We are saying that it participates in a Tat complex that is capable of transport, not that it will support transport.

2. The truncated form of Hcf106 interacts with the cpTat precursor as figure 7C. Does the truncated form of Hcf106 supports the Tat transport in thylakoid system?

We apologize for the confusion. Figure 7 uses full-length, Cys-substituted Hcf106. We showed Cys-substituted Hcf106 interacts with cpTatC and precursor strongly suggesting that cys-substituted Hcf106 alone could support transport. Testing the functionality of the truncated Hcf107 is currently under investigation.

All of these changes can be seen in the 'Tracked Changes' version of the manuscript that we are submitting along with a clean copy. Again, we thank the reviewers and editor for taking the time with our manuscript.

Sincerely,

Carole Dabney-Smith
Associate Professor, Chemistry and Biochemistry Department
